# A new implicit high-order iterative scheme for the numerical simulation of the two-dimensional time fractional Cable equation

**DOI:** 10.1038/s41598-023-28741-7

**Published:** 2023-01-27

**Authors:** Muhammad Asim Khan, Norma Alias, Ilyas Khan, Fouad Mohammad Salama, Sayed M. Eldin

**Affiliations:** 1 Department of Mathematical Sciences, Faculty of Science, Universiti Teknologi Malaysia, 81310 Johor Bahru, Johor Malaysia; 2 Department of Mathematics, College of Science Al-Zulfi, Majmaah University, Al-Majmaah, 11952 Saudi Arabia; 3 Department of Mathematics, King Fahd University of Petroleum and Minerals, Dhahran, Saudi Arabia; 4 Center of Research, Faculty of Engineering, Future University in Egypt, 11835 New Cairo, Egypt

**Keywords:** Applied mathematics, Computational science

## Abstract

In this article, we developed a new higher-order implicit finite difference iterative scheme (FDIS) for the solution of the two dimension (2-D) time fractional Cable equation (FCE). In the new proposed FDIS, the time fractional and space derivatives are discretized using the Caputo fractional derivative and fourth-order implicit scheme, respectively. Moreover, the proposed scheme theoretical analysis (convergence and stability) is also discussed using the Fourier analysis method. Finally, some numerical test problems are presented to show the effectiveness of the proposed method.

## Introduction

In the past few years, the popularity of fractional calculus increased due to its application in various branches of science and technology^1–5^. Many physical problems arise from different fields of sciences are mostly mathematically model using the fractional partial differential equations (FPDEs). These FPDEs are solved either using analytical or numerical methods but due to the complexity of FPDEs mostly it is difficult to solve using analytical methods^6–10^. Therefore, different numerical methods are used to solve these FPDEs e.g., finite difference, finite element, finite volume methods^6,9,11^. In these numerical methods, the finite difference method (FDM) is seen more in the literature because it is a simple and explicit method as compared to the other methods, especially the higher-order FDMs which converge fast as compared to the standard second-order FDMs.

In this article, the 2D-FCE is analyzed for the numerical solution using the higher-order FDM. The 2D-FCE is

1 $$\begin{aligned}_{0}^{C}\text {D}_{t}^{\gamma }w(x,y,t)&=\frac{\partial ^{2}w}{\partial x^{2}}+\frac{\partial ^{2}w}{\partial y^{2}} -\mu _{0}w(x,y,t) +g(x,y,t), \nonumber\\ (x,y)& \in (Q_1,Q_2)\times (Q_3,Q_4), ~~~t \in (0,T), \end{aligned}$$

where Caputo fractional derivative is represented by $_{0}^{C}\text {D}_{t}^{\gamma }w$, $(0<\gamma <1)$ and defined as^12^

$$\begin{aligned} _{0}^{C}D_{t}^{\gamma }w(x,y,t)=\frac{1}{\Gamma (1-\gamma )} \int _{0}^{t}\frac{w'(\tau )}{(t-\tau )^{\gamma }}d\tau , \end{aligned}$$

where $\Gamma (.)$ is Gamma function.

The FCE is modeled from Nernst–Planck equation or obtained from relating electrical properties in cell membrane and used for the approximation of complex microscopic motions of ions in nerve cells^13^. Throughout the course of recent years research on neuronal dendrites has increased^14^ because of the revelation that dendrites are profoundly dynamic, with complex electrical and bio-compound flagging relying upon both nearby spine structure and density^15^, and on voltage-gated particle channels^16^. These methods present challenges to the cable equation^17^. But due to the complexity of FCE various researchers solve FCE using different numerical methods, for instance, Liu et al.^18^ used implicit numerical method having second-order spatial accuracy for one dimensional (1-D) FCE. Similarly, Chen et al.^19^ solved 1-D variable-order FCE using the numerical method with higher-order spatial accuracy. Zhang et al.^20^ computed the numerical solution spline collocation methods for the 2-D FCE. They analyze the theoretical analysis (convergence and stability) is discussed using the Fourier analysis method. Furthermore, Balasim and Ali^21^ solved 2-D FCE using the implicit schemes having the spatial accuracy of second-order. Bhrawy and Zaky^22^ used spectral collocation method for both 1-D and 2-D FCE which is based on shifted Jacobi collocation method combined with the Jacobi operational matrix for fractional derivative. Ömer^23^ discussed the numerical solution for 2-D FCE using a meshless numerical method which is based on the hybridization of Gaussian and cubic kernels. Moreover, the FCE is solved on both regular and irregular domains. Nasrin and Abbas^24^ used the collocation numerical method for the solution of 1-D FCE where the proposed method is based on the combination of Bernoulli polynomials and Sinc functions which reduce the time FCE to the set of algebraic equations. Minghui et al.^25^ solved the FCE using local discontinuous Galerkin method in which the fractional time and spatial derivatives are discretized using the BDF2 with the L2 formula and local discontinuous Galerkin method, respectively. Ying and Lizhen^26^ used finite difference/spectral method for the numerical solution of generalized FCE in which backward difference and the Galerkin spectral methods are used for the time and space derivative, respectively. Also, the theoretical analysis (stability and convergence analysis) of proposed method is also analyzed which shows that the proposed method is unconditional stable and convergent. Xiaolin and Shuling^27^ proposed a mesh-less finite point method for the solution of FCE, in which moving least squares approximation and mesh-less smoothed gradient are combined with the proposed method to increase the rate of accuracy and convergence in space. Moreover the theoretical analysis of the proposed method are also discussed. However, the higher order numerical computationally efficient methods for the solution of the FCE are still in their early infancy. Therefore, the main objective of this article is to propose an unconditional stable and convergent higher order FDIS for the solution of 2-D FCE.

The content of the article is organized as follows; the proposed implicit numerical scheme development is discussed in “Formulation of the FDIS”, similarly, in “Stability” and “Convergence”, the theoretical analysis (stability and convergence) of the FDIS. The numerical examples are presented in “Numerical experiements”. Finally, the summary of the article is discussed in “Conclusion”.

## Formulation of the FDIS

To formulate the FDIS, the time and space dimensions are discretized as

$$\begin{aligned} \begin{aligned}x=ih_x, ~~~ y=jh_y,~~~ h_x=h_y=h=\frac{1}{m},~~~ t_k=\tau k,~~~ \tau =\frac{1}{n}~~~ \text {such that}, ~~~ i,j=0,1,2,\ldots ,m,~~~ k=0,1,2,\ldots ,n. \end{aligned} \end{aligned}$$

where time and space steps are represented by $\tau$ and *h* respectively. Let $\delta _x^2 w=w_{i+1,j}^k-2w_{i,j}^k +w_{i-1,j}^k$, then from Taylor series expansion

2 $$\begin{aligned}\delta _x^2 w_{i,j}^k= \frac{\partial ^2 w}{\partial x^2}|_{i,j}^{k} + \frac{h^2}{12}\frac{\partial ^4 w}{\partial x^4}|_{i,j}^{k} + \frac{h^4}{360}\frac{\partial ^6 w}{\partial x^6}|_{i,j}^{k} + O(h^6), \end{aligned}$$

3 $$\begin{aligned}\delta _y^2 w_{i,j}^k= \frac{\partial ^2 w}{\partial y^2}|_{i,j}^{k} + \frac{h^2}{12}\frac{\partial ^4 w}{\partial y^4}|_{i,j}^{k} + \frac{h^4}{360}\frac{\partial ^6 w}{\partial y^6}|_{i,j}^{k} + O(h^6). \end{aligned}$$

From (2) and (3)

4 $$\begin{aligned} \frac{\partial ^2 w}{\partial x^2}|_{i,j}^{k} = \left( 1+\frac{1}{12} \delta _x^2 \right) ^{-1} \frac{\delta _X^2}{h^2}w_{i,j}^k + O(h^4), \end{aligned}$$

5 $$\begin{aligned} \frac{\partial ^2 w}{\partial y^2}|_{i,j}^{k} = \left( 1+\frac{1}{12} \delta _y^2 \right) ^{-1} \frac{\delta _y^2}{h^2}w_{i,j}^k + O(h^4). \end{aligned}$$

The fractional discretization is^28^

6 $$\begin{aligned} \frac{\partial ^\gamma w(x_i,y_j,t_k)}{\partial t^\gamma }&=\frac{\tau ^{-\gamma }}{\Gamma (2-\gamma )}\sum _{r=0}^{k-1}b_r (w_{i,j}^{k-r}-w_{i,j}^{k-r-1}) + O(\tau ^ {2-\gamma }),\nonumber \\ b_r&=(r+1)^{1-\gamma }-r^{1-\gamma }, ~~~~r=\{0,1,2,\ldots \}. \end{aligned} $$

By using (4), (5) and (6) , the FCE become

$$\begin{aligned} & \frac{\tau ^{-\gamma }}{\Gamma (2-\gamma )}\sum _{r=0}^{k}b_r (w_{i,j}^{k+1-r}-w_{i,j}^{k-r}) = \left( 1+\frac{1}{12} \delta _x^2 \right) ^{-1} \frac{\delta _X^2}{h^2}w_{i,j}^{k+1} \\&\qquad +\left( 1+\frac{1}{12} \delta _y^2 \right) ^{-1} \frac{\delta _y^2}{h^2}w_{i,j}^{k+1} -\mu _{0} w_{i,j}^{k+1} + g_{i,j}^{k+1} + O(\tau ^{2-\gamma } +h^4), \\&\qquad \times \left( 1+\frac{1}{12}\delta _x^2\right) \left( 1+\frac{1}{12}\delta _y^2\right) \sum _{r=0}^k b_r(w_{i,j}^{k+1-r}-w_{i,j}^{k-r})\\&\quad =\frac{\tau ^\gamma \Gamma (2-\gamma )}{h^2} \left( \delta _x^2 +\delta _y^2 +\frac{1}{6}\delta _x^2 \delta _y^2 \right) w_{i,j}^{k+1} -\mu _{0} \tau ^\gamma \Gamma (2-\gamma ) \\&\qquad \times \left( 1+\frac{1}{12}\delta _x^2\right) w_{i,j}^{k+1}+\tau ^\gamma \Gamma (2-\gamma )\left( 1+\frac{1}{12}\delta _x^2\right) \left( 1+\frac{1}{12}\delta _y^2\right) g_{i,j}^{k+1}. \end{aligned}$$

After simplification, the FDIS is

7 $$\begin{aligned} \begin{aligned} a_0 w_{i,j}^{k+1}&= a_1 \left( w_{i+1,j}^{k+1}+ w_{i-1,j}^{k+1}+w_{i,j+1}^{k+1}+w_{i,j-1}^{k+1}\right) \\&\quad +a_2 \left( w_{i+1,j+1}^{k+1}+ w_{i-1,j+1}^{k+1}+w_{i+1,j-1}^{k+1}+w_{i-1,j-1}^{k+1}\right) \\&\quad + h^{2}\left( \frac{25}{36}w_{i,j}^{k} +\frac{5}{72}\left( w_{i+1,j}^{k}+w_{i-1,j}^{k}+w_{i,j+1}^{k}+w_{i,j-1}^{k}\right) \right. \\&\quad \left. +\frac{1}{144} \left( w_{i+1,j+1}^{k}+w_{i-1,j+1}^{k} + w_{i+1,j-1}^{k}+w_{i-1,j-1}^{k}\right) \right) + G_{i,j}^{k+1} \\ {}&\quad - h^2 \sum _{r=1}^{k}b_r \left( \frac{25}{36} w_{i,j}^{k+1-r}+ \frac{5}{72}\left( w_{i+1,j}^{k+1-r}+w_{i-1,j}^{k+1-r}+w_{i,j+1}^{k+1-r}+w_{i,j-1}^{k+1-r}\right) \right. \\&\quad \left. + \frac{1}{144}\left( w_{i+1,j+1}^{k+1-r}+w_{i-1,j+1}^{k+1-r}+w_{i-1,j+1}^{k+1-r}+w_{i-1,j-1}^{k+1-r}\right) \right. \\ {}&\quad -\left. \Bigg (\frac{25}{36} w_{i,j}^{k-r}+ \frac{5}{72}\left( w_{i+1,j}^{k-r}+w_{i-1,j}^{k-r}+w_{i,j+1}^{k-r}+w_{i,j-1}^{k-r}\right) \right. \\&\quad \left. + \frac{1}{144}\left( w_{i+1,j+1}^{k-r}+w_{i-1,j+1}^{k-r}+w_{i-1,j+1}^{k-r}+w_{i-1,j-1}^{k-r}\right) \Bigg )\right) , \end{aligned} \end{aligned}$$

where

$$\begin{aligned} \begin{aligned} & g_0=\tau ^\gamma \Gamma (2-\gamma ), a_0=\frac{10}{3}g_0 + \frac{25}{36} h^2(1+ g_0), a_1=\frac{2}{3}g_0 - \frac{5}{72} h^2(1+ g_0),\\ {}&a_2=\frac{g_0}{6} - \frac{1}{144} h^2(1+ g_0), b_r= (r+1)^{1-\gamma }-(r)^{1-\gamma }, ~~~\text {and} \\ {}&G_{i,j}^{k+1}= g_0 h^2\left( \frac{25}{36}g_{i,j}^{k+1} +\frac{5}{72}\Big(g_{i+1,j}^{k+1}+g_{i-1,j}^{k+1}+g_{i,j+1}^{k+1}+g_{i,j-1}^{k+1} +\frac{1}{144}\Big(g_{i+1,j+1}^{k+1}+g_{i-1,j+1}^{k+1}+g_{i+1,j-1}^{k+1}+g_{i-1,j-1}^{k+1}\Big)\right) . \end{aligned} \end{aligned}$$

**Figure 1:**
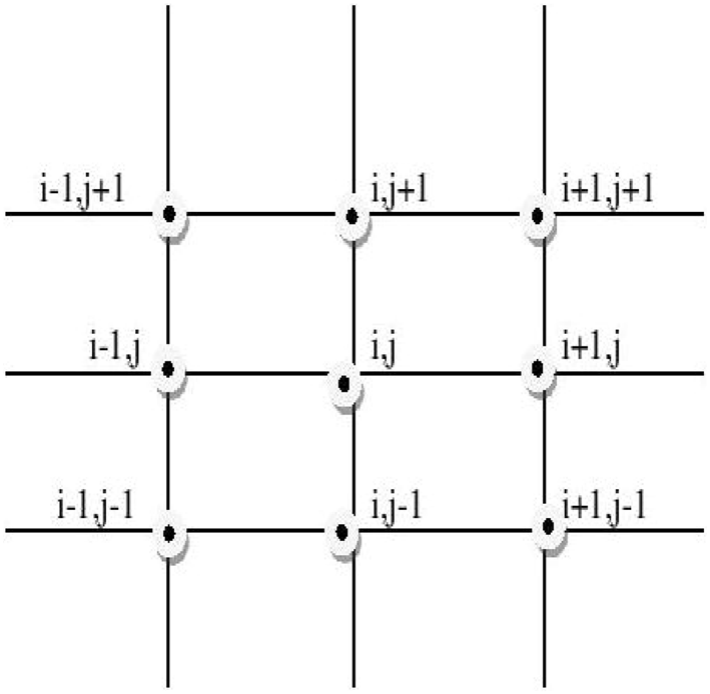
Grid points for the proposed scheme (8).

**Figure 2:**
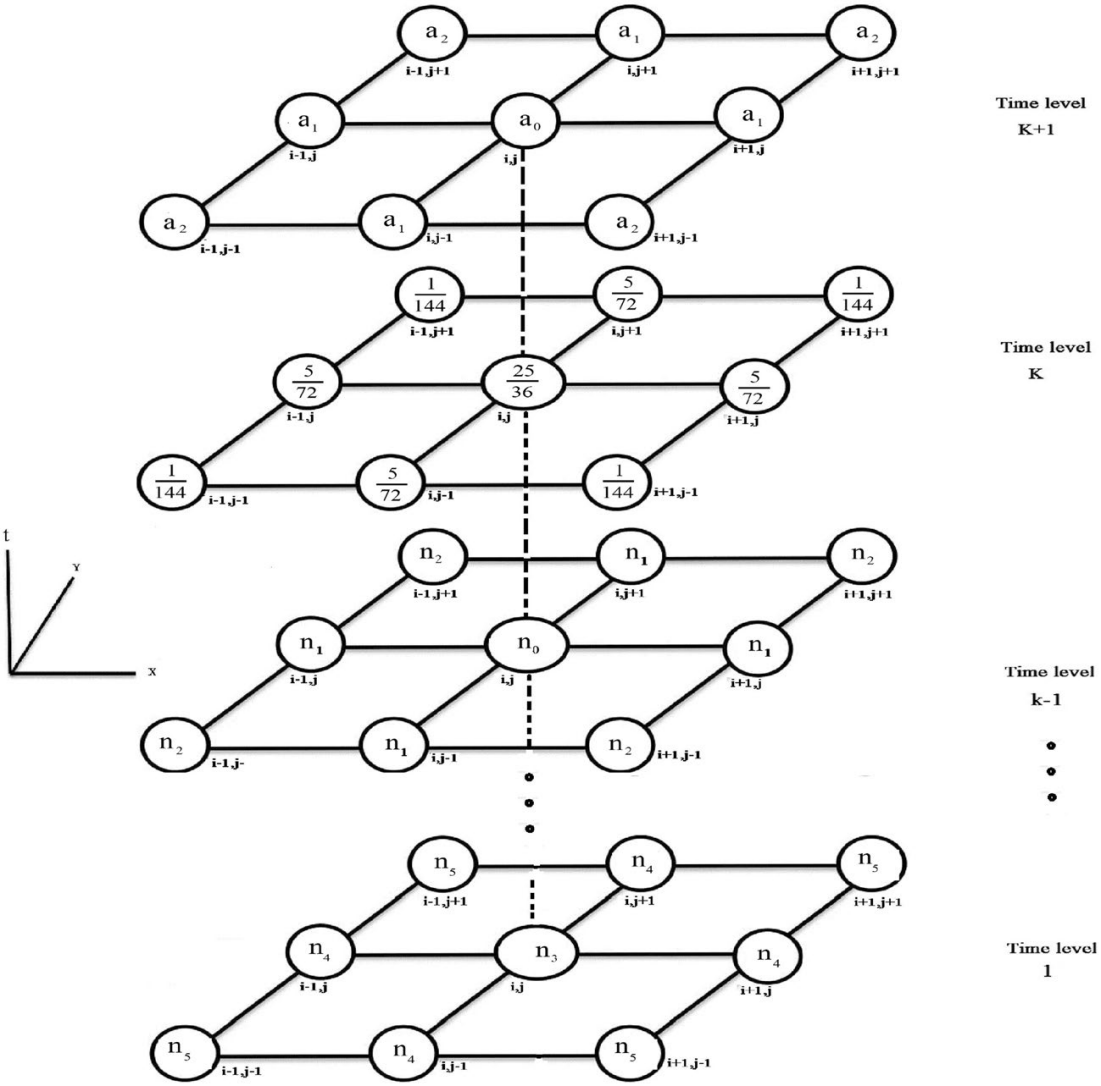
Computational molecule for the proposed scheme (7).

Figure 1 shows nine points on the grid, while In Fig. 2, the computational molecule of FDIS (7) is presented , where $n_{0}=\frac{25}{18}b_2g_0$, $n_1=\frac{5}{36}b_2g_0$, $n_2=\frac{1}{72}b_2g_0$, $n_3=\frac{25}{18}b_kg_0$, $n_4=\frac{5}{36}b_kg_0$ and $n_5 =\frac{1}{72}b_kg_0$.

## Stability

Let the approximate and exact solutions are presented by $w_{i,j}^{k}$ and $W_{i,j}^{k}$ for the FDIS (7), respectively, and $\vartheta _{i,j}^{k}= W_{i,j}^{k}-w_{i,j}^{k}$ then from (7) we get

8 $$\begin{aligned}a_0 \vartheta _{i,j}^{k+1}&= a_1 \Big( \vartheta _{i+1,j}^{k+1}+ \vartheta _{i-1,j}^{k+1}+\vartheta _{i,j+1}^{k+1}+\vartheta _{i,j-1}^{k+1}\Big) +a_2 \Big( \vartheta _{i+1,j+1}^{k+1}+ \vartheta _{i-1,j+1}^{k+1}+\vartheta _{i+1,j-1}^{k+1}+\vartheta _{i-1,j-1}^{k+1}\Big) \\ & \quad + h^{2}\Bigg[ \frac{25}{36}\vartheta _{i,j}^{k} +\frac{5}{72}\Big(\vartheta _{i+1,j}^{k}+\vartheta _{i-1,j}^{k}+\vartheta _{i,j+1}^{k}+\vartheta _{i,j-1}^{k}\Big) +\frac{1}{144} \Big(\vartheta _{i+1,j+1}^{k}+\vartheta _{i-1,j+1}^{k} + \vartheta _{i+1,j-1}^{k}+\vartheta _{i-1,j-1}^{k}\Big)\Bigg] \\ {}& \quad - h^2 \sum _{r=1}^{k}b_r \Bigg[ \frac{25}{36} \vartheta _{i,j}^{k+1-r}+ \frac{5}{72}\Big(\vartheta _{i+1,j}^{k+1-r}+\vartheta _{i-1,j}^{k+1-r}+\vartheta _{i,j+1}^{k+1-r}+\vartheta _{i,j-1}^{k+1-r}\Big) + \frac{1}{144}\Big(\vartheta _{i+1,j+1}^{k+1-r}+\vartheta _{i-1,j+1}^{k+1-r}+\vartheta _{i-1,j+1}^{k+1-r}+\vartheta _{i-1,j-1}^{k+1-r}\Big) \\& \quad - \Big( \frac{25}{36} \vartheta _{i,j}^{k-r}+ \frac{5}{72}\Big(\vartheta _{i+1,j}^{k-r}+\vartheta _{i-1,j}^{k-r}+\vartheta _{i,j+1}^{k-r}+\vartheta _{i,j-1}^{k-r}\Big) + \frac{1}{144}\Big(\vartheta _{i+1,j+1}^{k-r}+\vartheta _{i-1,j+1}^{k-r}+\vartheta _{i-1,j+1}^{k-r}+\vartheta _{i-1,j-1}^{k-r}\Big)\Bigg) \Bigg] , \end{aligned} $$

having initial and boundary conditions

9 $$\begin{aligned} \begin{aligned}\vartheta _{i,j}^{0}=\vartheta _{m,0}^{k}=\vartheta _{0,m}^{k}=0,\\ {}&\vartheta _{i,m}^{k}=\vartheta _{m,j}^{k}=0,~~i,j=1,2,\ldots ,m-1,~~ k=1,2,\ldots ,n-1. \end{aligned} \end{aligned}$$

The error function is described as

10 $$\begin{aligned} \vartheta ^{k}(x,y)=\left\{ \begin{matrix} \vartheta _{i,j}^{k} &{} \text {when} &{} x\in (x_{i-\frac{h}{2}}, x_{i+\frac{h}{2}}],~~ x\in (y_{i-\frac{h}{2}}, y_{i+\frac{h}{2}}],\\ 0 &{} \text {when} &{} x \in [0, \frac{h}{2}]~~ \text {or}~~x \in [L- \frac{h}{2}, L],\\ 0&{} \text {when} &{} y \in [0, \frac{h}{2}]~~ \text {or}~~L- y \in [\frac{h}{2}, L]. \end{matrix}\right. \end{aligned}$$

The error function $\vartheta _{i,j}^{k}$ in terms of Fourier series^29^

11 $$\begin{aligned} \vartheta ^{k}(x,y)=\sum _{l_{0},l_{1}=-\infty }^{\infty }\rho ^{k}(l_{0},l_{1})\exp \Bigg (2\sqrt{-1}\pi \Bigg (\frac{l_{0}x}{L_0}+\frac{l_{1}y}{L_0}\Bigg )\Bigg ), \end{aligned}$$

where

12 $$\begin{aligned} \rho ^{k}(l_{0},l_{1})=\frac{1}{L^2_0}\int _{0}^{L_0}\int _{0}^{L_0}\vartheta ^{k}(x,y) \exp \Bigg (-2\sqrt{-1}\pi \Bigg (\frac{l_{0}x}{L_0}+\frac{l_{1}y}{L_0}\Bigg )\Bigg )dxdy. \end{aligned}$$

The $l^{2}$-norm definition for $\vartheta _{i,j}^{k}$ is

13 $$\begin{aligned} \left\| \vartheta ^{k} \right\| _{l^{2}}^{2}=h^2\sum _{i=1}^{m}\sum _{j=1}^{m}\left| \vartheta _{i,j}^{k} \right| ^{2}. \end{aligned}$$

The relationship between Parseval equality and $l^{2}$-norm is

14 $$\begin{aligned} \left\| \vartheta ^{k} \right\| _{l^{2}}^{2}=\sum _{i=1}^{m}\sum _{j=1}^{m}h^2\left| \vartheta _{i,j}^{k} \right| ^{2}=\sum _{l_{0},l_{1}=-\infty }^{\infty }\left| \rho ^{k}(l_{0},l_{1}) \right| ^{2}. \end{aligned}$$

Suppose

15 $$\begin{aligned} \vartheta _{i,j}^{k}=\rho ^{k}e^{\sqrt{-1}(\varphi _{1}ih + \varphi _{2}jh)}, \end{aligned}$$

where $$\varphi _{1}=\frac{2\pi l_{0}}{L_0}$$, $$\varphi _{2}=\frac{2\pi l_{1}}{L_0}$$.

16 $$\begin{aligned} \begin{aligned}a_0 \rho ^{k+1} = a_1 \Big(\rho ^{k+1}e^{I(\varphi _{1}h)} + \rho ^{k+1}e^{-I(\varphi _{1}h)} + \rho ^{k+1}e^{I(\varphi _{2}h)} \\ {}&\quad +\rho ^{k+1}e^{-I(\varphi _{2}h)}\Big) + a_2 \Big(\rho ^{k+1}e^{I(\varphi _{1} + \varphi _{2})h} + \rho ^{k+1}e^{I(\varphi _{2} - \varphi _{1})h} + \rho ^{k+1}e^{I(\varphi _{1} - \varphi _{2})h}\\ {}&\quad + \rho ^{k+1}e^{I(-\varphi _{1} - \varphi _{2})h}\Big) + \frac{25h^2}{36} \rho ^{k} + \frac{5h^2}{72} \Big( \rho ^{k}e^{I(\varphi _{1}h)} \\ {}&\quad + \rho ^{k}e^{I(-\varphi _{1}h)} + \rho ^{k}e^{I( \varphi _{2}h)} + \rho ^{k}e^{I( -\varphi _{2}h)}\Big) + \frac{h^2}{144} ( \rho ^{k}e^{I\Big(\varphi _{1} + \varphi _{2})h} + \rho ^{k}e^{(I(\varphi _{2} -\varphi _{1})h}\\&\quad + \rho ^{k}e^{I(\varphi _{1} - \varphi _{2})h} + \rho ^{k}e^{I(-\varphi _{1} - \varphi _{2})h}\Big) - h^{2}\sum _{r=1}^{k}b_r \left( \frac{25}{36} \rho ^{k+1-r} + \frac{5}{72}(\rho ^{k+1-r}e^{I(\varphi _{1}h)} \right. \\ {}&\quad \left. + \rho ^{k+1-r}e^{I(-\varphi _{1}h )} + \rho ^{k+1-r}e^{I( \varphi _{2}h)} +\rho ^{k+1-r}e^{I(-\varphi _{2}(h)}|Bigg) + \frac{1}{144}\Big( \rho ^{r+1}e^{I(\varphi _{1} + \varphi _{2})h} \right. \\ {}&\quad \left. + \rho ^{k+1-r}e^{I(\varphi _{2} - \varphi _{1})h} + \rho ^{k+1-r}e^{I(\varphi _{1} - \varphi _{2})h} + \rho ^{k+1-r}e^{I(-\varphi _{1} - \varphi _{2})h}\Big) - \Bigg ( \frac{25}{36} \rho ^{k-r} + \frac{5}{72}\Big(\rho ^{k-r}e^{I(\varphi _{1}h)} \right. \\ {}&\quad \left. + \rho ^{k-r}e^{I(-\varphi _{1}h )} + \rho ^{k-r}e^{I( \varphi _{2}h)} +\rho ^{k-r}e^{I(-\varphi _{2}(h)}\Big) + \frac{1}{144}\Big( \rho ^{k-r}e^{I(\varphi _{1} + \varphi _{2})h} \right. \\ {}&\quad \left. + \rho ^{k-r}e^{I(\varphi _{2} - \varphi _{1})h} + \rho ^{k-r}e^{I(\varphi _{1} - \varphi _{2})h} + \rho ^{k-r}e^{I(-\varphi _{1} - \varphi _{2})h}\Big)\Bigg ) \right) . \end{aligned} \end{aligned}$$

By using Euler’s formula for exponential function

17 $$\begin{aligned} \begin{aligned}e^{I(\varphi _{1}h)}+e^{-I(\varphi _{1}h)}+ e^{I( \varphi _{2}h)} + e^{-I( \varphi _{2}h)}=2( \cos (\varphi _{1}h) + \cos (\varphi _{2}h))\\ {}&\text {and} \\ {}&e^{I(\varphi _{1} + \varphi _{2})h} + e^{I(\varphi _{2} - \varphi _{1})h} + e^{I(\varphi _{1} - \varphi _{2})h} +e^{I(-\varphi _{1} - \varphi _{2})h}= 4\cos (\varphi _{1}h) \cos ( \varphi _{2}h). \end{aligned} \end{aligned}$$

Substituting Eq. (17) in Eq. (16), we have

18 $$\begin{aligned} \begin{aligned} a_0 \rho ^{k+1}&= 2a_1 \rho ^{k+1}( \cos (\varphi _{1}h) +\cos ( \varphi _{2}h)) + 4a_2 \rho ^{k+1}\cos (\varphi _{1}h)\cos (\varphi _{2}h) \\ {}&\quad + \frac{25h^2}{36} \rho ^{k} + \frac{5h^2}{36} \rho ^{k}( \cos (\varphi _{1}h) + \cos (\varphi _{2}h)) + \frac{h^2}{36}\rho ^{k} \cos (\varphi _{1}h)\cos (\varphi _{2}h) \\ {}&\quad - \sum _{r=1}^{k-1}(a_{k-r+1}-a_{k-r}) \left( \frac{25h^{2}}{36}\Big (\rho ^{k+1-r}-\rho ^{k-r}\Big) + \frac{5h^{2}}{36}\Big(\rho ^{k+1-r}-\rho ^{k-r}\Big)( \cos (\varphi _{1}h) + \cos (\varphi _{2}h)) \right. \\ {}&\quad \left. + \frac{h^{2}}{36} \Big(\rho ^{k+1-r}-\rho ^{k-r}\Big)\cos (\varphi _{1}h)\cos (\varphi _{2}h)\right) . \end{aligned} \end{aligned}$$

Then simplifying Eq. (18) for $\rho ^{k+1}$

19 $$\begin{aligned} \begin{aligned} \rho ^{k+1}&= \frac{h^2}{36}\Bigg ( \frac{25+5m_0 + m_1}{a_0 - 2a_1 m_0 -4a_2 m_1}\Bigg )\rho ^{k}\\ {}&\quad - \frac{h^{2}}{36} \sum _{r=1}^{k}b_r \left( \Bigg (\frac{ 25+5m_0 + m_1}{a_0 - 2a_1 m_0 -4a_2 m_1}\Bigg )\rho ^{k+1-r} -\frac{ 25+5m_0 + m_1}{a_0 - 2a_1 m_0 -4a_2 m_1}\Bigg )\rho ^{k-r} \right) , \end{aligned} \end{aligned}$$

where $$m_0=\cos (\varphi _{1} h) +\cos ( \varphi _{2}h)$$ and $$m_1=\cos (\varphi _{1}h)\cos ( \varphi _{2}h)$$.

### Proposition 1

Suppose $\rho ^{k+1}$ satisfies (19), then $\left| \rho ^{k+1} \right| \le \left| \rho ^{0}\right|$.

### Proof

When $k=0$, then from (19)

20 $$\begin{aligned} \begin{aligned}\rho ^{1} = \frac{h^2}{36}\left( \frac{25+5m_0 + m_1}{a_0 - 2a_1 m_0 -4a_2 m_1}\right) \rho ^{0}. \end{aligned} \end{aligned}$$

Since, the maximum value of $\cos (x)$ is 1, therefore

21 $$\begin{aligned} \begin{aligned}|\rho ^{1}| \le \left( \frac{1}{1+g_0} \right) |\rho ^{0}|, \end{aligned} \end{aligned}$$

where $$g_0=\tau ^\gamma \Gamma (2-\gamma )>0$$. therefore

$$\begin{aligned} \left| \rho ^{1} \right| \le \left| \rho ^{0} \right| . \end{aligned}$$

Let

22 $$\begin{aligned} \left| \rho ^{m} \right| \le \left| \rho ^{0} \right| ; ~~~~~~ m=1,2,\ldots ,k, \end{aligned}$$

then for $$m=k+1$$

23 $$\begin{aligned} \begin{aligned} \rho ^{k+1}&= \frac{h^2}{36}\Bigg ( \frac{25+5m_0 + m_1}{a_0 - 2a_1 m_0 -4a_2 m_1}\Bigg )\rho ^{k}\\ {}&\quad - \frac{h^{2}}{36} \sum _{r=1}^{k}b_r \left( \left( \frac{ 25+5m_0 + m_1}{a_0 - 2a_1 m_0 -4a_2 m_1}\right) \rho ^{k+1-r} -\left( \frac{ 25+5m_0 + m_1}{a_0 - 2a_1 m_0 -4a_2 m_1}\right) \rho ^{k-r} \right) . \end{aligned} \end{aligned}$$

Taking absolute function on both sides

$$\begin{aligned} \begin{aligned}\left| \rho ^{k+1} \right| \le \frac{h^2}{36} \left| \frac{25+5m_0 + m_1}{a_0 - 2a_1 m_0 - 4a_2 m_1}\right| \left| \rho ^{k} \right| + \frac{h^2}{36}\left| \frac{25+5m_0 + m_1}{a_0 - 2a_1 m_0 - 4a_2 m_1}\right| \sum _{r=1}^{k} b_r\left( |\rho ^{k+1-r}-\rho ^{k-r}| \right) , \end{aligned} \end{aligned}$$

using (22)

$$\begin{aligned} \begin{aligned}\left| \rho ^{k+1} \right| \le \frac{h^2}{36} \left| \frac{25+5m_0 + m_1}{a_0 - 2a_1 m_0 - 4a_2 m_1}\right| |\left( 1+ (b_1 +b_2 +\cdots +b_k)|\right) |\rho ^{0}|, \end{aligned} \\ \begin{aligned}\left| \rho ^{k+1} \right| \le \frac{h^2}{36} \left| \frac{25+5m_0 + m_1}{a_0 - 2a_1 m_0 - 4a_2 m_1}\right| \left( |2(k+1)^{1-\gamma }-1|\right) |\rho ^{0}| \end{aligned} \end{aligned}$$

Substituting the values of $m_0$ and $m_1$, and after simplifying we get

$$\begin{aligned} \begin{aligned}\left| \rho ^{k+1} \right| \le \frac{2((k+1)^{1-\gamma }-1)}{ (1 + g_0)} |\rho ^{0}|, \end{aligned} \end{aligned}$$

if $g_0 \ge 2(k+1)^{1-\gamma }$, then $0< \frac{2h^2((k+1)^{1-\gamma }-1)}{24g_0 +11h^2 (1+g_0)} \le 1$, thus

$$\begin{aligned} \begin{aligned}\left| \rho ^{k+1} \right| \le |\rho ^{0}|. \end{aligned} \end{aligned}$$

Hence

$$\begin{aligned} ||\rho ^{k+1} || \le ||\rho ^{0} ||. \end{aligned}$$

Therefore, the numerical solution satisfies

$$\begin{aligned} ||\vartheta ^{k+1} || \le ||\vartheta ^{0} ||. \end{aligned}$$

$$\square $$

## Convergence

Suppose $\Re ^{k+1}$ represents the truncation error at $w(x_{i},y_{i},t_{k+1})$, then

$$\begin{aligned} \begin{aligned} \Re ^{k+1}&=\frac{\tau ^{-\gamma }}{\Gamma (2-\gamma )}\sum _{r=0}^{k}b_r (w_{i,j}^{k+1-r}-w_{i,j}^{k-r})-\left( 1+\frac{1}{12} \delta _x^2 \right) ^{-1} \frac{\delta _X^2}{h^2}w_{i,j}^{k+1}\\&\quad - \left( 1+\frac{1}{12} \delta _y^2 \right) ^{-1} \frac{\delta _y^2}{h^2}w_{i,j}^{k+1} -U_0 w_{i,j}^{k+1} - g_{i,j}^{k+1}\\ {}&=\frac{\tau ^{-\gamma }}{\Gamma (2-\gamma )}\sum _{r=0}^{k}b_r (w_{i,j}^{k+1-r}-w_{i,j}^{k-r}) - \frac{\partial ^\gamma w}{\partial t^\gamma }|_{i,j}^{k+1} +\frac{\partial ^2 w}{\partial x^2} |_{i,j}^{k+1}\\&\quad -\left( 1+\frac{1}{12} \delta _x^2 \right) ^{-1} \frac{\delta _X^2}{h^2}w_{i,j}^{k+1} + \frac{\partial ^2 w}{\partial y^2} |_{i,j}^{k+1} \\ {}&\quad -\left( 1+\frac{1}{12} \delta _y^2 \right) ^{-1} \frac{\delta _y^2}{h^2}w_{i,j}^{k+1} \\ {}&=\frac{\tau ^{-\gamma }}{\Gamma (2-\gamma )}\sum _{r=0}^{k}b_r (w_{i,j}^{k+1-r}-w_{i,j}^{k-r}) - \frac{\partial ^\gamma w}{\partial t^\gamma }|_{i,j}^{k+1} + \left[ \frac{\partial ^2 w}{\partial x^2} |_{i,j}^{k+1}-\left( 1+\frac{1}{12} \delta _x^2 \right) ^{-1} \frac{\delta _X^2}{h^2}w_{i,j}^{k+1} \right] \\ {}&\quad + \left[ \frac{\partial ^2 w}{\partial y^2} |_{i,j}^{k+1} -\left( 1+\frac{1}{12} \delta _y^2 \right) ^{-1} \frac{\delta _y^2}{h^2}w_{i,j}^{k+1} \right] , \\ {}&= O (\tau ^{2-\gamma }) -\left( \frac{h^4}{360} \frac{\partial ^6 u}{\partial x^6} +\cdots \right) - \left( \frac{h^4}{360} \frac{\partial ^6 u}{\partial y^6} +\cdots \right) \\ {}&=O(\tau ^{2-\gamma } + h^{4} ) \end{aligned} \end{aligned}$$

24 $$\begin{aligned} \left| \Re ^{k+1} \right| \le f_1(\tau ^{2-\gamma } + h^{4} ), \end{aligned}$$

where $f_1$ is a constant. Let $\vartheta _{i,j}^{k}=W_{i,j}^{k}-w_{i,j}^{k}$, where *W* and *w* represent the exact and approximate respectively, then from Eq. (7)

25 $$\begin{aligned} \begin{aligned}a_0 \vartheta _{i,j}^{k+1}&= a_1 \left( \vartheta _{i+1,j}^{k+1}+ \vartheta _{i-1,j}^{k+1}+\vartheta _{i,j+1}^{k+1}+\vartheta _{i,j-1}^{k+1}\right) +a_2 \left( \vartheta _{i+1,j+1}^{k+1}+ \vartheta _{i-1,j+1}^{k+1}+\vartheta _{i+1,j-1}^{k+1}+\vartheta _{i-1,j-1}^{k+1}\right) \\ {}&\quad + h^{2}\left( \frac{25}{36}\vartheta _{i,j}^{k} +\frac{5}{72}\left( \vartheta _{i+1,j}^{k}+\vartheta _{i-1,j}^{k}+\vartheta _{i,j+1}^{k}+\vartheta _{i,j-1}^{k}\right) +\frac{1}{144} \left( \vartheta _{i+1,j+1}^{k}+\vartheta _{i-1,j+1}^{k} + \vartheta _{i+1,j-1}^{k}+\vartheta _{i-1,j-1}^{k}\right) \right) \\ {}&\quad - h^2 \sum _{r=1}^{k}b_r \left( \frac{25}{36} \vartheta _{i,j}^{k+1-r}+ \frac{5}{72}\left( \vartheta _{i+1,j}^{k+1-r}+\vartheta _{i-1,j}^{k+1-r} +\vartheta _{i,j+1}^{k+1-r}+\vartheta _{i,j-1}^{k+1-r}\right) \right. \\ {}&\quad \left. + \frac{1}{144}\Big(\vartheta _{i+1,j+1}^{k+1-r}+\vartheta _{i-1,j+1}^{k+1-r} +\vartheta _{i-1,j+1}^{k+1-r}+\vartheta _{i-1,j-1}^{k+1-r}\Big) \right. \\&\quad -\left. \Bigg (\frac{25}{36} \vartheta _{i,j}^{k-r}+ \frac{5}{72}\left( \vartheta _{i+1,j}^{k-r}+\vartheta _{i-1,j}^{k-r}+\vartheta _{i,j+1}^{k-r}+\vartheta _{i,j-1}^{k-r}\right) \right. \\&\quad \left. +\frac{1}{144}\left( \vartheta _{i+1,j+1}^{k-r}+\vartheta _{i-1,j+1}^{k-r}+\vartheta _{i-1,j+1}^{k-r} +\vartheta _{i-1,j-1}^{k-r}\right) \Bigg )\right) +\Re ^{k+1}, \end{aligned} \end{aligned}$$

with initial and boundary conditions

26 $$\begin{aligned}& \vartheta _{i,j}^{0}=\vartheta _{m,0}^{k}=\vartheta _{0,m}^{k}=0,\\ &\vartheta _{i,m}^{k}=\vartheta _{m,j}^{k}=0,  \end{aligned}$$

$$ i,j=1,2,\ldots ,m-1,~~ k=1,2,\ldots ,n-1.$$

Define the truncation error function $$ R^{k}(x,y)$$ as,

$$\begin{aligned} \Re ^{k}(x,y)=\left\{ \begin{matrix} \Re _{i,j}^{k} &{} when &{} x \in (x_{i-\frac{h}{2}}, x_{i+\frac{h}{2}}],~~ x \in (y_{i-\frac{h}{2}}, y_{i+\frac{h}{2}}],\\ 0&{} when &{} x \in [0, \frac{h}{2}],~~ x \in [L-\frac{h}{2}, L],\\ 0 &{} when &{} y \in [0, \frac{h}{2}],~~ y \in [L-\frac{h}{2}, L]. \end{matrix}\right. \end{aligned}$$

Express $\vartheta ^{k}$ and $\Re ^{k}$ functions as Fourier series

27 $$\begin{aligned} \vartheta _{i,j}^{k}= & {} \rho ^{k}e^{I(\varphi _{1} i h+ \varphi _{2} j h)}, \; I=\sqrt{-1}, \end{aligned}$$

28 $$\begin{aligned} \Re _{i,j}^{k}= & {} \mu ^{k}e^{I(\varphi _{1} i h + \varphi _{2} j h)}, \; I=\sqrt{-1}, \end{aligned}$$

where $$\varphi _{1}=\frac{2\pi l_{1}}{L}$$, $$\varphi _{2}=\frac{2\pi l_{2}}{L}$$.

Substituting (27) and (28) into (25), we have

29 $$\begin{aligned} \begin{aligned}a_0 \rho ^{k+1} = a_1 \Big(\rho ^{k+1}e^{I(\varphi _{1}h)} + \rho ^{k+1}e^{-I(\varphi _{1}h)} + \rho ^{k+1}e^{I(\varphi _{2}h)} \\&\quad +\rho ^{k+1}e^{-I(\varphi _{2}h)}\Big) + a_2 \Big(\rho ^{k+1}e^{I(\varphi _{1} + \varphi _{2})h} + \rho ^{k+1}e^{I(\varphi _{2} - \varphi _{1})h} + \rho ^{k+1}e^{I(\varphi _{1} - \varphi _{2})h}\\ {}&\quad + \rho ^{k+1}e^{I(-\varphi _{1} - \varphi _{2})h}\Big) + \frac{25h^2}{36} \rho ^{k} + \frac{5h^2}{72} \Big( \rho ^{k}e^{I(\varphi _{1}h)} \\ {}&\quad +\rho ^{k}e^{I(-\varphi _{1}h)} + \rho ^{k}e^{I( \varphi _{2}h)} + \rho ^{k}e^{I( -\varphi _{2}h)}\Big) + \frac{h^2}{144} ( \rho ^{k}e^{I(\varphi _{1} + \varphi _{2})h} + \rho ^{k}e^{(I(\varphi _{2} -\varphi _{1})h}\\ {}&\quad + \rho ^{k}e^{I(\varphi _{1} - \varphi _{2})h} + \rho ^{k}e^{I(-\varphi _{1} - \varphi _{2})h}\Big) - h^{2}\sum _{r=1}^{k}b_r \left( \frac{25}{36} \rho ^{k+1-r} + \frac{5}{72}\Big(\rho ^{k+1-r}e^{I(\varphi _{1}h)} \right. \\ {}&\quad \left. + \rho ^{k+1-r}e^{I(-\varphi _{1}h )} + \rho ^{k+1-r}e^{I( \varphi _{2}h)} +\rho ^{k+1-r}e^{I(-\varphi _{2}(h)}\Big) + \frac{1}{144}( \rho ^{r+1}e^{I(\varphi _{1} + \varphi _{2})h} \right. \\ {}&\quad \left. + \rho ^{k+1-r}e^{I(\varphi _{2} - \varphi _{1})h} + \rho ^{k+1-r}e^{I(\varphi _{1} - \varphi _{2})h} + \rho ^{k+1-r}e^{I(-\varphi _{1} - \varphi _{2})h}\Big) - \Bigg ( \frac{25}{36} \rho ^{k-r} + \frac{5}{72}(\rho ^{k-r}e^{I(\varphi _{1}h)} \right. \\ {}&\quad \left. + \rho ^{k-r}e^{I(-\varphi _{1}h )} + \rho ^{k-r}e^{I( \varphi _{2}h)} +\rho ^{k-r}e^{I(-\varphi _{2}(h)}\Big) + \frac{1}{144}\Big( \rho ^{k-r}e^{I(\varphi _{1} + \varphi _{2})h} \right. \\ {}&\quad \left. + \rho ^{k-r}e^{I(\varphi _{2} - \varphi _{1})h} + \rho ^{k-r}e^{I(\varphi _{1} - \varphi _{2})h} + \rho ^{k-r}e^{I(-\varphi _{1} - \varphi _{2})h}\Big)\Bigg ) \right) +\mu ^{k+1}. \end{aligned} \end{aligned}$$

Substituting (17) into (29), we get

30 $$\begin{aligned} \begin{aligned} a_0 \rho ^{k+1}&= 2 a_1 \rho ^{k+1}( \cos (\varphi _{1}h) +\cos ( \varphi _{2}h)) + 4 a_2 \rho ^{k+1}\cos (\varphi _{1}h)\cos (\varphi _{2}h) \\ {}&\quad + \frac{25h^2}{36} \rho ^{k} + \frac{5h^2}{36} \rho ^{k}( \cos (\varphi _{1}h) + \cos (\varphi _{2}h)) + \frac{h^2}{36}\rho ^{k} \cos (\varphi _{1}h)\cos (\varphi _{2}h) \\ {}&\quad - \sum _{r=1}^{k-1}(a_{k-r+1}-a_{k-r}) \left( \frac{25h^{2}}{36} (\rho ^{k+1-r}-\rho ^{k-r}) + \frac{5h^{2}}{36}(\rho ^{k+1-r}-\rho ^{k-r})( \cos (\varphi _{1}h) + \cos (\varphi _{2}h)) \right. \\ {}&\quad \left. + \frac{h^{2}}{36} (\rho ^{k+1-r}-\rho ^{k-r})\cos (\varphi _{1}h)\cos (\varphi _{2}h\right) +\mu ^{k+1}. \end{aligned} \end{aligned}$$

Simplifying (30) for $\rho ^{k+1}$, we obtain

31 $$\begin{aligned} \begin{aligned} \rho ^{k+1}&= \frac{h^2}{36}\Bigg ( \frac{25+5m_0 + m_1}{a_0 - 2a_1 m_0 -4a_2 m_1}\Bigg )\rho ^{k}\\&- \frac{h^{2}}{36} \sum _{r=1}^{k}b_r \left( \Bigg (\frac{ 25+5m_0 + m_1}{a_0 - 2a_1 m_0 -4a_2 m_1}\Bigg )\rho ^{k+1-r} -\frac{ 25+5m_0 + m_1}{a_0 - 2a_1 m_0 -4a_2 m_1}\Bigg )\rho ^{k-r} \right) +\frac{\mu ^{k+1}}{a_0 - 2a_1 m_0 -4a_2 m_1}. \end{aligned} \end{aligned}$$

### Proposition 2

Let $\rho ^{k+1}$ satisfies (31), then $\left| \rho ^{k+1} \right| \le \left| \mu ^{k+1}\right|$ where $k=0,1,2,\ldots ,n-1$.

### Proof

We know from (9) and (11)

32 $$\begin{aligned} \rho ^{0}=\rho ^{0}(l_{1},l_{2})=0. \end{aligned}$$

From (24)

33 $$\begin{aligned} |\mu ^{s+1}|\le |\mu |, ~~~~\forall ~~~~ s=\{0,2,\ldots k-1\}. \end{aligned}$$

When $$k=0$$ in (31)

34 $$\begin{aligned} \rho= & {} \frac{\mu }{a_0 - 2a_1 m_0 -4a_2 m_1}, \end{aligned}$$

35 $$\begin{aligned} \left| \rho \right|= & {} \frac{1}{|a_0 - 2a_1 m_0 -4a_2 m_1|} \left| \mu \right| , ~~~~~~ \because \text {taking absolute} \end{aligned}$$

36 $$\begin{aligned} \left| \rho \right|= & {} \frac{1}{h^2(1+g_0)} \left| \mu \right| , \end{aligned}$$

but $$h^2(1+g_0) >0$$, so

$$\begin{aligned} \left| \rho \right| \le \left| \mu \right| . \end{aligned}$$

Suppose

37 $$\begin{aligned} \left| \rho ^{s} \right| \le \left| \mu ^{s} \right| , ~~~~ \forall ~~~~s=\{1,2,\ldots ,k\}. \end{aligned}$$

From (30)

$$\begin{aligned} \begin{aligned} |\rho ^{k+1}|&= \Bigg |\frac{h^2}{36}\Bigg ( \frac{25+5m_0 + m_1}{a_0 - 2a_1 m_0 -4a_2 m_1}\Bigg )\rho ^{k}\\ {}&\quad - \frac{h^{2}}{36} \sum _{r=1}^{k}b_r \left( \Bigg (\frac{ 25+5m_0 + m_1}{a_0 - 2a_1 m_0 -4a_2 m_1}\Bigg )\rho ^{k+1-r} -\frac{ 25+5m_0 + m_1}{a_0 - 2a_1 m_0 -4a_2 m_1})\rho ^{k-r} \right) +\frac{\mu ^{k+1}}{a_0 - 2a_1 m_0 -4a_2 m_1}\Bigg |. \end{aligned} \end{aligned}$$

By using (37) and (33)

$$\begin{aligned} \begin{aligned} |\rho ^{k+1}|&\le \frac{2((k+1)^{1-\beta } -1 ) +36/h^2 }{1+g_0}|\mu ^{k+1}|. \end{aligned} \end{aligned}$$

If $g_0 \ge 2(k+1)^{1-\gamma } +36/h^2 -1$ then $0<\frac{2((k+1)^{1-\beta } -1 ) +36/h^2 }{1+g_0}\le 1$, hence

$$\begin{aligned} \left| \rho ^{k+1} \right| \le \left| \mu ^{k+1} \right| . \end{aligned}$$

Hence proof. $$\square $$

Now from (24) and (14), we have

38 $$\begin{aligned} \left\| \Re ^{k +\frac{1}{2}} \right\| \le Mhf_1(\tau ^{2-\gamma }+h^{4})=Lf_1(\tau ^{2-\gamma }+h^{4}). \end{aligned}$$

Using Proposition 2, and (14)

$$\begin{aligned} \parallel \vartheta ^{k+1}\parallel \le \parallel \Re ^{k+\frac{1}{2}} \parallel \le Lf_1(\tau ^{2-\gamma }+h^{4}), \\ \parallel \vartheta ^{k+1}\parallel \le f_1 L(\tau ^{2-\gamma } +h^{4}), \end{aligned}$$

hence, we get

39 $$\begin{aligned} \parallel \vartheta ^{k+1}\parallel \le T(\tau ^{2-\gamma } +h^{4}), \end{aligned}$$

where $$T= f_1 L$$.

Hence, the FDIS (7) is conditionally convergent with convergence order $O(\tau ^{2-\gamma }+h^{4})$.

## Numerical experiements

In current section, two examples are discussed to confirm the effectiveness of the FDIS for 2D FCE. In the proposed iterative method combined method is executed over the different time and mesh sizes. The numerical simulation is done using the PC with 4GB RAM, core i3, Windows 7, 3.40 GHz, and Mathematica software. The numerical examples are performed with the tolerance ($\omega$) for the maximum error $(l_\infty )$. The proposed method convergence orders are found using the following formula^30^.

$$\begin{aligned} \Im _{1}{\text {-}}order= & {} log_{2}\left( \frac{\parallel L_{\infty }(2\tau ,h) \parallel }{\parallel L_{\infty }(\tau ,h) \parallel }\right) , \\ \Im _2{\text {-}}order= & {} Log_2\left( \frac{||l_\infty (16\tau ,2h)||}{||l_\infty (\tau ,h)||} \right) . \end{aligned}$$

### Example 1

Consider the model problem^31^

$$\begin{aligned} _0^C D_t^\gamma w(x,y,t)= \frac{\partial ^2w}{\partial x^2} +\frac{\partial ^2w}{\partial y^2} -u + \left( \frac{2}{\Gamma (3-\gamma )}t^{2-\gamma }+t^2(1+2\pi ^2) \right) \sin (\pi x)\sin (\pi y), \end{aligned}$$

having analytical solution

$$\begin{aligned} w(x,y,t)=t^{2} \sin (\pi x)\sin (\pi y). \end{aligned}$$

### Example 2

Consider the model problem^22^

$$\begin{aligned} _{0}^{c} D_{t}^{\gamma } u=\frac{\partial ^{2} u}{\partial x^{2}}+\frac{\partial ^{2} u}{\partial y^{2}}-u+e^{x+y}\left( \frac{2 t^{2-\gamma }}{\Gamma (3-\gamma )}-t^{2}\right) , \end{aligned}$$

having analytical solution

$$\begin{aligned} u(x, y, t)=t^{2} e^{x+y}. \end{aligned}$$

**Figure 3:**
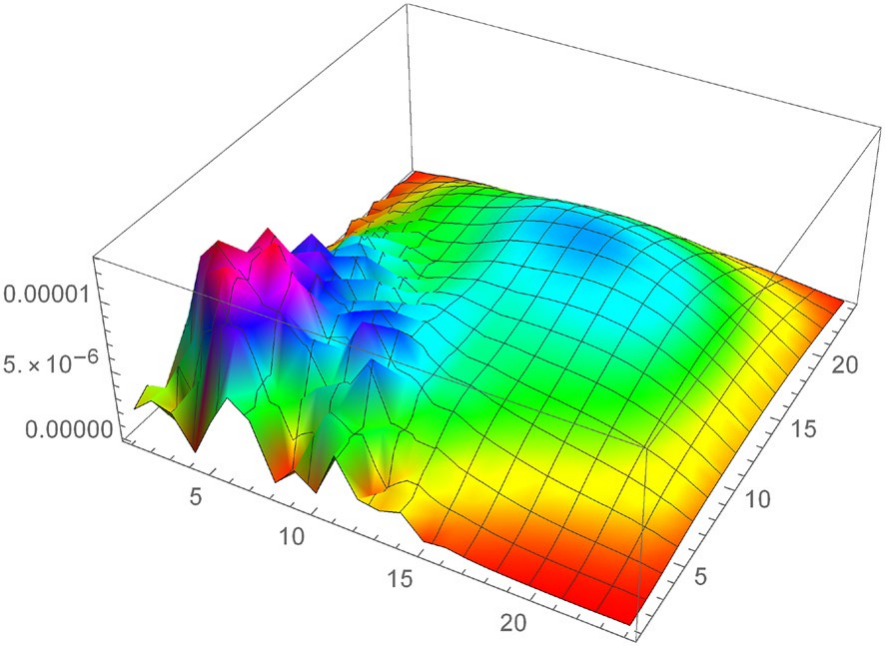
The Example 1 absolute error, when $h=\tau =\frac{1}{25}$ and $\gamma =0.1$.

**Figure 4:**
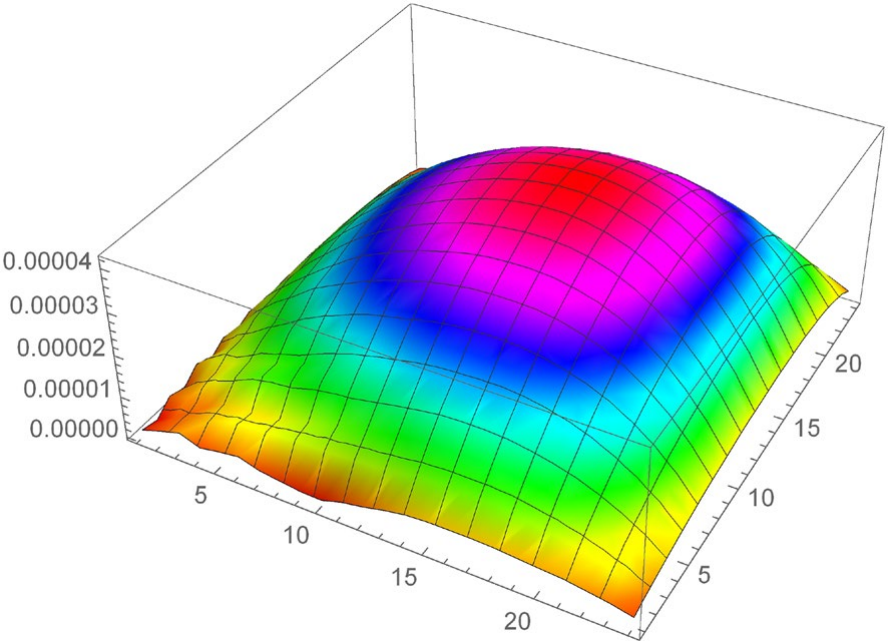
The Example 2 absolute error when $h=\tau =\frac{1}{25}$ and $\gamma =0.1$.

**Figure 5:**
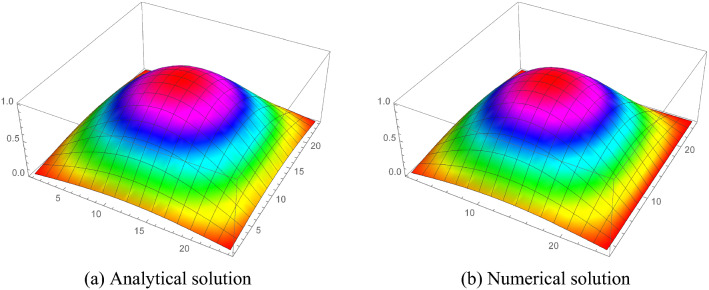
Analytical and numerical solution for example 1, when $h=\tau =\frac{1}{25}$.

**Figure 6:**
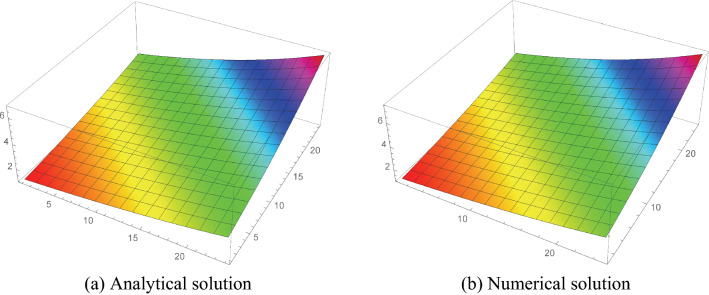
Analytical and numerical solution for example 1, when $h=\tau =\frac{1}{25}$.

Tables 1, 2, 3 and 4 numerical results shows that the errors (maximum error ”M_E”, average error ”A_E”) are reduced with decreasing mesh size. Also, Tables 5 and 6 show that the proposed method gives better results as compared to the^32^ and^20^, which shows the effectiveness of the proposed method. Furthermore, in Tables 7 and 8, the spatial variable order of convergence is presented for different values of $\gamma$ which depict the spatial variable order of convergence in agreement with the theoretical spatial accuracy of the proposed scheme for examples 1 and 2. Similarly, Tables 9 and 10 consist of the temporal variable order of convergence for the different values of $\gamma$ which show that the theoretical and experimental temporal variable convergence accuracy is also in agreement. Moreover, the graphical representation in 3-D graphs of the proposed scheme is presented in Figs. 2, 3, 4, 5 and 6, which affirms FDIS effectiveness.

**Table 1:** Numerical results for the Example 1, where $\gamma = 0.1$.

$\tau$	*h*	Iteration	M_E	A_E
$\frac{1}{5}$	$\frac{1}{5}$	44	$6.9757 \times 10^{-4}$	$4.5350 \times 10^{-4}$
$\frac{1}{10}$	$\frac{1}{10}$	40	$8.9688 \times 10^{-5}$	$4.3799 \times 10^{-5}$
$\frac{1}{15}$	$\frac{1}{15}$	40	$3.2365 \times 10^{-5}$	$1.5531 \times 10^{-6}$
$\frac{1}{20}$	$\frac{1}{20}$	42	$1.6872 \times 10^{-5}$	$8.2234 \times 10^{-6}$
$\frac{1}{30}$	$\frac{1}{30}$	40	$4.3668 \times 10^{-5}$	$9.0917 \times 10^{-6}$

**Table 2:** Numerical results for the Example 1, where $\gamma = 0.5$.

$\tau$	*h*	Iteration	M_E	A_E
$\frac{1}{5}$	$\frac{1}{5}$	45	$2.1487 \times 10^{-3}$	$7.2797 \times 10^{-5}$
$\frac{1}{10}$	$\frac{1}{10}$	30	$6.8921 \times 10^{-3}$	$1.4044 \times 10^{-3}$
$\frac{1}{15}$	$\frac{1}{15}$	40	$3.5734 \times 10^{-4}$	$3.3747 \times 10^{-4}$
$\frac{1}{20}$	$\frac{1}{20}$	44	$1.0562 \times 10^{-4}$	$2.3659 \times 10^{-4}$
$\frac{1}{30}$	$\frac{1}{30}$	40	$9.3649 \times 10^{-5}$	$6.0262 \times 10^{-5}$

**Table 3:** Numerical results for the Example 2, where $\gamma = 0.1$.

$\tau$	*h*	Iteration	M_E	A_E
$\frac{1}{5}$	$\frac{1}{5}$	52	$7.0901 \times 10^{-4}$	$4.6764 \times 10^{-5}$
$\frac{1}{10}$	$\frac{1}{10}$	52	$2.1412 \times 10^{-4}$	$1.1986 \times 10^{-4}$
$\frac{1}{15}$	$\frac{1}{15}$	48	$1.0695 \times 10^{-4}$	$5.4370 \times 10^{-5}$
$\frac{1}{20}$	$\frac{1}{20}$	54	$6.3901 \times 10^{-5}$	$3.2475 \times 10^{-5}$
$\frac{1}{30}$	$\frac{1}{30}$	65	$3.0330 \times 10^{-5}$	$1.5141 \times 10^{-5}$

**Table 4:** Numerical results for the Example 2, where $\gamma = 0.5$.

$\tau$	*h*	Iteration	M_E	A_E
$\frac{1}{5}$	$\frac{1}{5}$	53	$7.5742 \times 10^{-3}$	$5.0433 \times 10^{-3}$
$\frac{1}{10}$	$\frac{1}{10}$	53	$2.7558 \times 10^{-3}$	$1.5618 \times 10^{-3}$
$\frac{1}{15}$	$\frac{1}{15}$	48	$1.5230 \times 10^{-3}$	$8.1342 \times 10^{-4}$
$\frac{1}{20}$	$\frac{1}{20}$	55	$9.9445 \times 10^{-4}$	$5.1590 \times 10^{-4}$
$\frac{1}{30}$	$\frac{1}{30}$	65	$5.4703 \times 10^{-4}$	$2.7411 \times 10^{-4}$

**Table 5:** Comparison of the proposed scheme Eq. (7) with^32^ and^20^ for the Example 1, where $\gamma = 0.5$.

$\tau$	*h*	M_E	^32^	^20^
$\frac{1}{5}$	$\frac{1}{5}$	$2.1487 \times 10^{-3}$	$8.8496 \times 10^{-3}$	$3.8921 \times 10^{-2}$
$\frac{1}{10}$	$\frac{1}{10}$	$6.8921 \times 10^{-4}$	$2.2508 \times 10^{-3}$	$1.4625 \times 10^{-2}$
$\frac{1}{20}$	$\frac{1}{20}$	$2.3659 \times 10^{-4}$	$5.8320 \times 10^{-4}$	$5.3241 \times 10^{-3}$
$\frac{1}{30}$	$\frac{1}{30}$	$1.3649 \times 10^{-4}$	$2.6199 \times 10^{-4}$	$2.9349 \times 10^{-3}$

**Table 6:** Comparison of the proposed scheme Eq. (7) with^32^ and^20^ for the Example 1, where $\gamma = 0.6$.

$\tau$	*h*	M_E	^32^	^20^
$\frac{1}{5}$	$\frac{1}{5}$	$2.8893 \times 10^{-3}$	$9.2673 \times 10^{-3}$	$9.7966 \times 10^{-3}$
$\frac{1}{10}$	$\frac{1}{10}$	$1.0360 \times 10^{-3}$	$2.3281 \times 10^{-3}$	$3.2412 \times 10^{-3}$
$\frac{1}{20}$	$\frac{1}{20}$	$3.8988 \times 10^{-4}$	$5.9869 \times 10^{-4}$	$1.0054 \times 10^{-3}$
$\frac{1}{30}$	$\frac{1}{30}$	$2.2034 \times 10^{-4}$	$2.6804 \times 10^{-4}$	$5.0562 \times 10^{-4}$

**Table 7:** Space variable convergence order for the Example 1.

$h/\tau$	$\gamma =0.1$		$\gamma =0.25$	
	M_E	$\Im _2$-order	M_E	$\Im _2$-order
$h=\tau =\frac{1}{2}$	$2.6172 \times 10^{-2}$	–	$2.7699\times 10^{-2}$	–
$h=\frac{1}{4},\tau =\frac{1}{32}$	$1.4687 \times 10^{-3}$	4.38	$1.4820 \times 10^{-3}$	4.25
$h=\tau =\frac{1}{4}$	$1.7141 \times 10^{-3}$	–	$2.2530 \times 10^{-3}$	–
$h=\frac{1}{8},\tau =\frac{1}{64}$	$8.7578 \times 10^{-5}$	4.70	$9.0833 \times 10^{-4}$	4.33
	$\gamma =0.5$		$\gamma =0.75$	
$h/\tau$	M_E	$\Im _2$-order	M_E	$\Im _2$-order
$h=\tau =\frac{1}{2}$	3.1809 $\times 10^{-2}$	–	$3.8695 \times 10^{-2}$	–
$h=\frac{1}{4},\tau =\frac{1}{32}$	1.5616 $\times 10^{-3}$	4.34	3.9160 $\times 10^{-3}$	4.43
$h=\tau =\frac{1}{4}$	$1.8538 \times 10^{-3}$	–	$1.0527 \times 10^{-2}$	–
$h=\frac{1}{8},\tau =\frac{1}{64}$	1. 2965 $\times 10^{-4}$	4.91	$2.7630 \times 10^{-4}$	4.38

**Table 8:** Space variable convergence order for the Example 2.

$h/\tau$	$\gamma =0.25$		$\gamma =0.5$	
	M_E	$\Im _2$-order	M_E	$\Im _2$-order
$h=\tau =\frac{1}{2}$	$1.00571 \times 10^{-2}$	–	$2.7664\times 10^{-2}$	–
$h=\frac{1}{4},\tau =\frac{1}{32}$	$6.0059 \times 10^{-4}$	4.06	$8.7258 \times 10^{-4}$	4.98
$h=\tau =\frac{1}{4}$	$3.2472 \times 10^{-3}$	–	$1.0060 \times 10^{-2}$	–
$h=\frac{1}{8},\tau =\frac{1}{64}$	$9.3560 \times 10^{-5}$	5.11	$1.7833 \times 10^{-4}$	5.81
	$\gamma =0.75$		$\gamma =0.9$	
$h/\tau$	M_E	$\Im _2$-order	M_E	$\Im _2$-order
$h=\tau =\frac{1}{2}$	5.6346 $\times 10^{-2}$	–	8.0570 $\times 10^{-2}$	–
$h=\frac{1}{4},\tau =\frac{1}{32}$	1.7955 $\times 10^{-3}$	4.97	3.8020 $\times 10^{-3}$	4.40
$h=\tau =\frac{1}{4}$	2.3634 $\times 10^{-2}$	–	3.7141 $\times 10^{-2}$	–
$h=\frac{1}{8},\tau =\frac{1}{64}$	7. 727 $\times 10^{-4}$	4.93	1.8113 $\times 10^{-3}$	4.35

**Table 9:** Temporal variable convergence order for the Example 1, when $h=\frac{1}{8}$.

$\tau$	$~~~~~~~~\gamma =0.75$		$~~~~~~~~\gamma =0.9$	
	$~~~~~L_{\infty }$	$\Im _1$-Order	$~~~~~L_{\infty }$	$\Im _1$-Order
$\tau =\frac{1}{10}$	$1.9523 \times 10^{-3}$	–	$3.4198 \times 10^{-3}$	–
$\tau =\frac{1}{20}$	8.7950 $\times 10^{-4}$	1.15	1.6413 $\times 10^{-3}$	1.05
$\tau =$ $\frac{1}{40}$	4.2234 $\times 10^{-4}$	1.05	8.1259 $\times 10^{-4}$	1.01
$\tau =\frac{1}{80}$	$2.3416 \times 10^{-4}$	0.85	$4.1987\times 10^{-4}$	0.95

**Table 10:** Temporal variable convergence order for the Example 2, when $h=\frac{1}{8}$Temporal variable convergence order for the Example 2, when $h=\frac{1}{8}$.

$\tau$	$~~~~~~~~\gamma =0.5$		$~~~~~~~~\gamma =0.9$	
	$~~~~~L_{\infty }$	$\Im _1$-Order	$~~~~~L_{\infty }$	$\Im _1$-Order
$\tau =\frac{1}{10}$	$2.7254 \times 10^{-3}$	–	$1.3956\times 10^{-2}$	–
$\tau =\frac{1}{20}$	9.879 $\times 10^{-4}$	1.46	6.5285 $\times 10^{-3}$	1.09
$\tau =$ $\frac{1}{40}$	3.5286 $\times 10^{-4}$	1.48	3.0520 $\times 10^{-3}$	1.09
$\tau =\frac{1}{80}$	$1.3016 \times 10^{-4}$	1.43	$1.4177 \times 10^{-3}$	1.10

## Conclusion

The higher-order FDIS is established and analyzed for the 2-D FCE. The theoretical analysis of the proposed method shows that the proposed method is unconditionally stable and convergent with the fourth-order of convergence. Moreover, the proposed method is reliable and effective for the numerical solutions of 2-D FCE. Furthermore, The proposed method’s theoretical convergence order is $O(\tau ^{2-\gamma } +h^4)$, and $C_{2}$-order shows that the theoretical and numerical spatial order of convergence is in agreement.

## Data Availability

The data presented in this work is available from the corresponding author on reasonable request.
